# Laboratory Evaluation of Geosynthetic Interface Friction under Low Stress

**DOI:** 10.3390/polym16172519

**Published:** 2024-09-05

**Authors:** Paolo Carrubba

**Affiliations:** Department of Civil, Environmental and Architectural Engineering, University of Padua, 35131 Padua, Italy; paolo.carrubba@unipd.it

**Keywords:** geosynthetics, interface strength, mode of failure

## Abstract

In landfill cover, geosynthetic packages are often used to fulfil different and simultaneous functions: drainage, waterproofing, separation, reinforcement, and soil protection. In this regard, various types of geosynthetics are combined in succession to allow for water and biogas drainage and to waterproof, reinforce, and provide protection from erosion over the useful lifetime, ranging over many decades if we consider the long phases of disposal, closure, and quiescence of the landfill itself. The creation of the composite cover barrier requires the evaluation of various interfaces’ frictional strength under low contact stresses, both in static and seismic cases. The main purpose of this study is to summarize the results of past laboratory tests carried out on different geosynthetic–geosynthetic and geosynthetic–soil–geosynthetic interfaces using experimental instrumentation developed at the geotechnical laboratory of the University of Padua, which allows for the characterization of the interface geosynthetic friction at low contact stresses. The main aspects highlighted are the kinematic mode of failure, the wearing of the contact surfaces, the presence or absence of interstitial fluid, and, finally, the density level of the granular soil in contact with the geosynthetics.

## 1. Introduction

Geosynthetics are widely used in landfill cover barriers since they are able to simultaneously guarantee the drainage of biogas and rainwater in their respective ascending and descending flows, waste waterproofing against fluid emission and entry, and cover reinforcement to provide stability on slopes and/or to absorb tensile forces induced by differential settlements. Finally, some geosynthetics are also able to prevent the erosion of the shallower soil exposed to atmospheric agents.

- Drainage functions are guaranteed by drainage geocomposites, which offer a continuous low-friction surface for the filtering geosynthetics. An appropriate filter opening is required in order to prevent unwanted long-term phenomena, such as blinding and clogging, both of which are capable of negating the drainage properties of the geocomposite itself.
- The waterproofing of both fluid emission and entry is achieved with the aid of a geomembrane, possibly coupled with a geocomposite clay liner or with a clay layer. To protect the geomembrane, a geonet is placed above it with the aim of avoiding possible lacerations during the subsequent phase of laying the drainage geocomposite.

These types of geosynthetics also offer continuous and/or discontinuous surfaces with low friction, for example, in the contact between the geonet and geomembrane, the geonet and drainage geocomposite, and the geomembrane and geocomposite clay liner.

- If the covering lies on a slope and/or in areas where substantial differential settlements are expected, a geogrid is inserted into the covering soil to increase strength to slippage and to relieve the induced tensile forces in the less resistant geosynthetics.

In order to protect the drainage geocomposite from mechanical damage during the construction phase, it may be necessary to insert a geogrid or a reinforced geomat immediately over the drainage geocomposite. However, strengthening the covering package’s resistance to tensile actions involves the introduction of further low-friction surfaces subjected to low contact stress.

- Geocells or geomats can be placed to prevent the soil erosion exposed to atmospheric agents and also to allow for natural greening or hydro-seeding.

**Figure 1:**
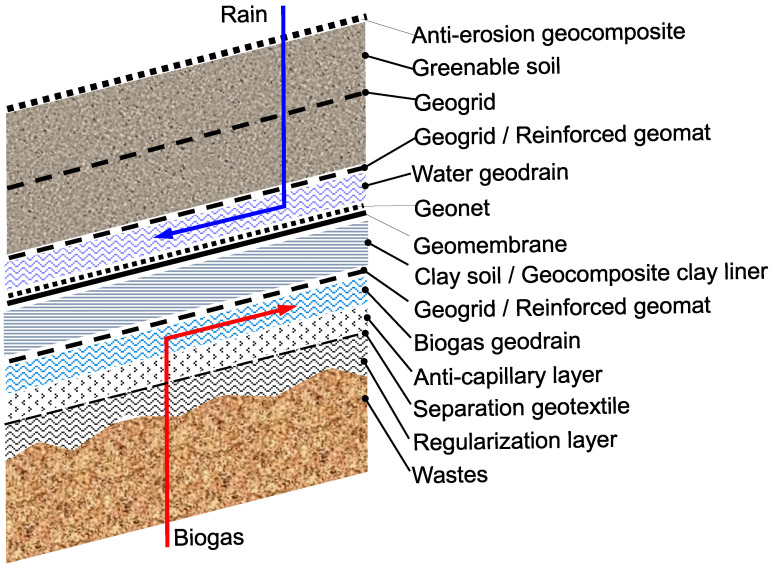
Schematic representation of a geosynthetic succession in a sloping landfill cover.

A typical succession of geosynthetics for building landfill covers on slopes is shown in Figure 1, from a qualitative point of view. Polymers typically used in these geosynthetic packages include polypropylene (PP), which is widely used to produce woven and nonwoven geotextiles, geodrains, geomats, and geocomposites. Polyethylene (PE) is generally used in the production of geocells and geomats for protecting the soil from erosion, while high-density polyethylene (HDPE) is used in the production of extruded geomembranes and geonets, as well as in the production of geogrids for soil reinforcement. Polyester (PET) is also used to manufacture high-tenacity geogrids and to coat steel geogrids.

The main purpose of this study is to summarize the results of past laboratory tests carried out on different geosynthetic–geosynthetic and geosynthetic–soil–geosynthetic interfaces using experimental instrumentation developed at the geotechnical laboratory of the University of Padua. This instrumentation consists of an inclined plane device and a direct shear device at a constant rate of loading. Both devices allow for the characterization of the geosynthetic interface friction under low contact stresses, and since they provide perfectly comparable results, they can be considered complementary and interchangeable, benefitting the test modalities that may be required in the characterization of the geosynthetic interface strength. The results of these experiments confirm how the interface friction of a landfill cover is a crucial parameter for the stability of the cover itself. The main aspect is the kinematic mode of failure, a function of the types of geosynthetics placed in contact, but also worthy of consideration are the state of wear of the contact surfaces, the presence or absence of interstitial fluid, and, finally, the density level of the granular soil in contact with the geosynthetics.

## 2. Laboratory Measurement of the Interface Strength under Low Stress

The interface shear strength between geosynthetics can be evaluated in the laboratory with different types of tests focusing on static, cyclic, and seismic conditions, as well as tests for low or high contact stresses. With respect to static tests conducted under low contact stress, the most frequently encountered tests in the technical literature are as follows: the direct shear test at constant displacement speed, the direct shear test at constant load speed, and the inclined plane test.

- The standard direct shear test at constant displacement speed is perhaps the most widespread in the technical literature and has been the subject of standardization [1,2]. It is performed on samples with a minimum size of 0.30 × 0.30 m, fixed horizontally to two supports, one of which remains fixed while the other can slide relative to the first. Once a predetermined normal pressure has been applied, σ_0_, the mobile box is made to slide during the test at a constant speed, and the shear stress, τ_0_, as a reaction to the impressed displacements, is measured.

**Figure 2:**
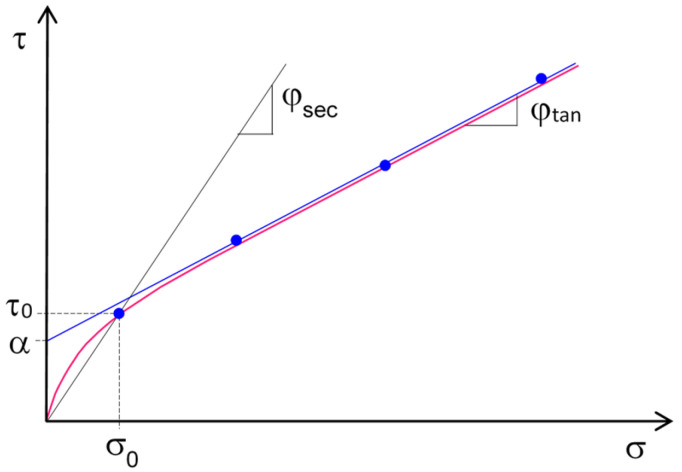
Definition of tangent (φ_tan_ and α) and secant (φ_sec_) strength parameters from the results of direct shear tests at low contact stress.

In order to identify the failure envelope, the tests are repeated for at least three different values of normal stress σ (which are, according to the European standard, 50 kPa, 100 kPa, and 150 kPa), and the shear strength parameters—adhesion, α, and slope of the envelope, φ_tan_—are evaluated via linear interpolation of the results. In this regard, it should be noted that, in the transition from low to high contact stresses, the failure envelope of the geosynthetic interfaces can be strongly curvilinear [3,4], such that the evaluation of the secant friction angles, φ_sec_, instead of the tangent, proves to be more in line with the actual performance of the cover package, since the normal stress level can be well below 50 kPa (Figure 2).

**Figure 3:**
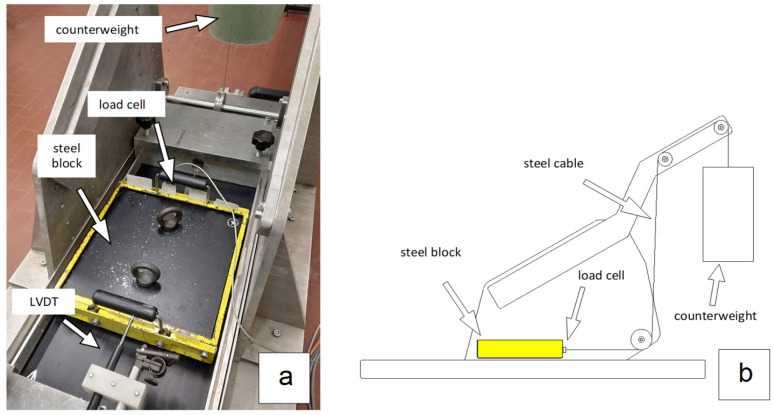
Direct shear device at constant loading speed. The device was made at the geotechnical laboratory of the University of Padua for the study of interfaces subjected to low normal stress: (**a**) top view; (**b**) operational scheme.

- A prototype of a direct shear device with constant speed of loading has been made at the geotechnical laboratory of the University of Padua [5,6]. Figure 3a shows a top view of the prototype, while Figure 3b shows the device’s operational scheme. It consists of a steel box with sides of 0.30 × 0.30 m placed on a horizontal plane. The first geosynthetic specimen is fixed on the base of the mobile box, while the second is fixed on the base of the device.

The box is connected, via a steel cable, to a counterweight and a load cell, placed between the box and the cable, which allows the horizontal force acting on the box to be measured during the test. The counterweight is increased over time with a constant speed of loading until the box reaches a maximum displacement (of about 50 mm). In cases where stress is applied statically, the mobilized friction angle can be evaluated using the following expression:

(1) $$tan\varphi =\frac{H}{W}$$

where

*H* is the horizontal force applied to the box;

*W* is the weight of the box itself.

An important difference between the standard direct shear test at constant displacement speed and the direct shear test at constant loading speed concerns the value of the minimum normal stress; due to the way in which the stress is applied in the standard direct shear test, by using a hydraulic press, the minimum contact stresses that can be reached are roughly 20 kPa. Instead, the prototype direct shear device at constant loading rate can perform tests at normal stresses as low as 5 kPa. 

**Figure 4:**
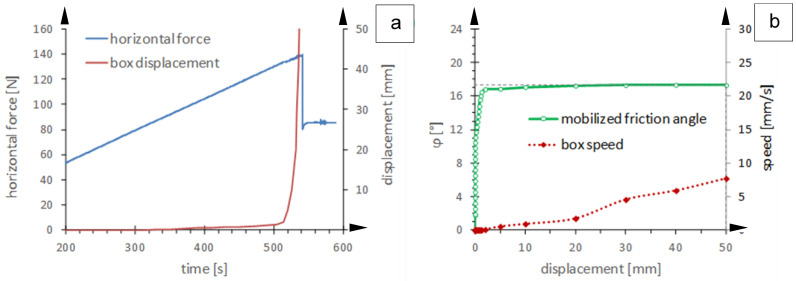
Results of a direct shear test at constant loading speed for an interface between a smooth geomembrane and a drainage geocomposite [5]: (**a**) evolution of the horizontal force and box displacements over time; (**b**) angle of mobilized friction and velocity of the box as a function of the displacement of the box itself.

Figure 4 shows a typical result of a direct shear test at constant loading speed for an interface of a smooth geomembrane and a drainage geocomposite using the experimental equipment of the geotechnical laboratory of the University of Padua [5].

Referring to the differences between the standard direct shear test and the direct shear test with constant loading speed, it should be noted that in the standard device, the displacement increases at a constant speed of about 1 mm/min, and the reaction of the interface is detected in terms of mobilized friction at the contact surface. Conversely, in the experimental device at constant loading speed, the shear stress is gradually increased through the counterweight, and the relative displacement of the interface is detected. The latter approach is more in line with the progressive loading of an interface on a slope, as well as with the loading mode followed with the inclined plane test and discussed below.

A further possibility offered by direct shear tests, including both standard ones and ones with a controlled speed of loading, is the option to carry out the shear tests in the presence of interstitial fluid, a circumstance not possible in the subsequent inclined plane test, where, at best, only the wet conditions can be examined.

**Figure 5:**
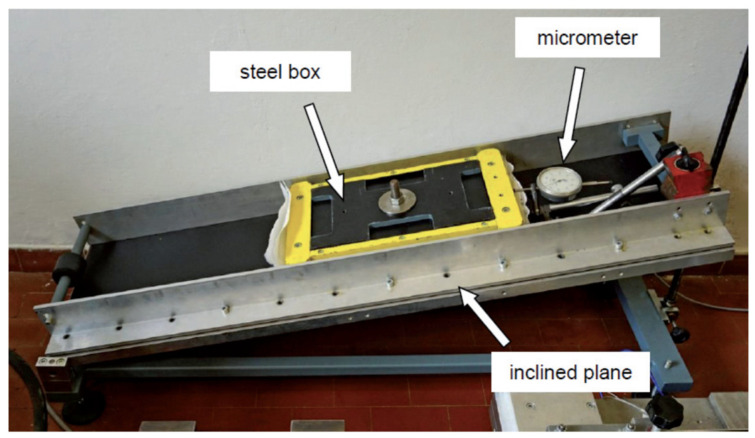
Inclined plane equipment available at the geotechnical laboratory of the University of Padua.

- The inclined plane test [7,8,9] has been standardized according to the European standard [10]. Strictly speaking, this standard refers only to interfaces between soil and geosynthetics, but its indications can also be easily extended to cases featuring contact between geosynthetics. The equipment made at the geotechnical laboratory of the University of Padua (Figure 5) consists of a tilting plane, above which, a steel box that is free to slide along the plane is placed. The motion of the box can be controlled via lateral guides and trolleys positioned outside the contact zone of the geosynthetics.

One geosynthetic of the interface is fixed on the inclined plane, while the other one is fixed to the bottom of the box. According to the standard [10], the specimen to be tested should have a minimum dimension of 0.30 × 0.30 m, analogous to the standard for the direct shear test. The test starts from the horizontal position of the plane; then, the table inclination is gradually increased with a constant speed of 3 ± 0.5°/min.

The plane inclination, β_50_, for which the box reaches 50 mm in displacement, must be identified; under this condition, the interface strength angle, φ_stand_, is evaluated with the following expression:

(2) $$tan\varphi_{stand}=tan\beta_{50}$$

Although not expressly specified in [10], it is possible to define a first interface strength angle for a minimum displacement of the box of, for example, 1 mm.

Under this hypothesis, a first detachment friction angle, φ_0_, may be defined when the plane inclination, β_0_, allows the box to reach a displacement of 1 mm. The first detachment friction angle is given by the following:

(3) $$tan\varphi_{0}=tan\beta_{0}$$

It should be noted that the displacement of 50 mm actually constitutes an arbitrary reference since Equation (2) is based on the hypothesis that the box is fixed and, therefore, in static equilibrium while it is moving with speed and acceleration that are not always negligible [11].

In this regard, various types of motion are possible:

- The sudden sliding mode is defined as that for which, once φ_0_ is reached, the box goes forward with uniformly accelerated motion (a_box_ = cost) and the friction strength becomes dynamic. It can be evaluated by means of the dynamic equilibrium equation, in which both the box accelerations and the gravity, g, are included:

(4) $$tan\varphi_{dyn}=tan\beta -\frac{1}{cos\beta }\;\frac{a_{box}}{g}$$

also, $\beta \geq \beta_{0}$

Therefore, within the standard relating to the inclined plane, the evaluation of the friction parameter through Equation (2) represents, in this circumstance, an index parameter and not a dynamic one; the latter can be obtained from Equation (4) if box acceleration is detected during the sliding phase.

- The gradual sliding mode is defined as one in which, once φ_0_ is reached, the box goes forward with uniform motion (a_box_ = 0), whose speed, v, becomes a function of the plane inclination, β(t), when the latter exceeds the value β_0_; in this case, a part of the reaction to the box motion, τ, comes from the viscous resistance (τ = c × v). Under the hypothesis that the speed remains constant in correspondence with the table inclination β(t), a kinematic strength angle, φ_kin_, is provided by the following expression:

(5) $$tan\varphi_{kin}\left(\beta \right)=tan\beta \;$$

also, $\beta >\beta_{0}$.

**Figure 6:**
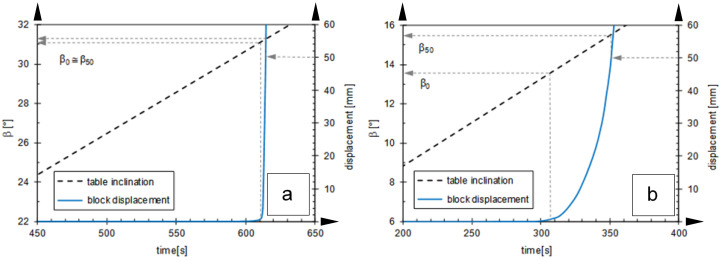
Two different HDPE geomembranes in contact with a nonwoven geotextile under dry conditions [12]: (**a**) the interface with the GMB_text_-GC D shows a sudden sliding behaviour; (**b**) the GMB_smooth_-GCD interface shows a gradual sliding behaviour.

To illustrate the diversity with which friction is mobilized between geosynthetics during inclined plane tests, some typical interface behaviours are proposed in Figure 6 [12]. The first case (Figure 6a) reflects the behaviour of an interface between a rough geomembrane (GMB_text_) and a drainage geocomposite (GCD) and shows a sudden sliding behaviour: φ_0_ is reached for very small displacements, less than a millimetre, after which the almost instantaneous slippage of the box occurs. In this case, β_0_ ≅ β_50_.

Contrastingly, in the case of Figure 6b, relating to the coupling of a smooth geomembrane (GMB_smooth_) with the same GCD as that of the previous case, a succession of kinematic equilibria are established until the conventional collapse inclination, β_50_, with movements progressively increasing in speed as long as β increases beyond β_0_. In this case, β_0_ < β_50_.

For this second interface, which can be classified in the gradual sliding mode, the evaluation of the mobilized friction at β_50_ is not very significant, since the first detachment of the interface occurs at the inclination, β_0_, i.e., in the absence of viscous resistance to motion.

- The conventional angle of interface strength, φ_stand_, is more significant for geosynthetic interfaces showing non-uniform motion during failure. For example, a rough geomembrane in contact with the nonwoven geotextile of a drainage geocomposite can give rise to considerable displacements well beyond β_50_ due to the effect of interpenetration of the geotextile fibres within the roughness of the geomembrane; for these interfaces, φ_0_ cannot be significant. Interfaces of particular polymers and geometries can manifest irregular motion during slip, as in the case of the stick slip mode of failure. It is a kinematic phenomenon characterized by sudden accelerations and stops as soon as the abrupt transition from static to dynamic friction occurs. The resulting motion is an alternating of stick and slip phases, which do not allow for a failure point to be identified.
- In addition to the standard test, described earlier, the inclined plane device allows for the evaluation of the interface friction by means of a further procedure, namely the force method [13].

**Figure 7:**
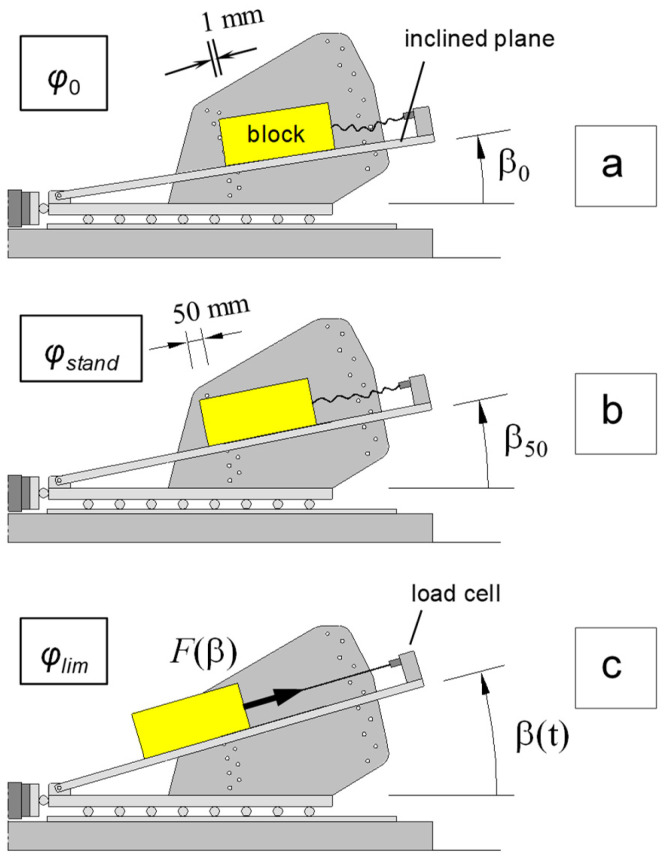
Complete procedure for measuring all the geosynthetic interface strengths with the inclined plane test: (**a**) friction angle at detachment, φ_0_; (**b**) standard friction angle, φ_stand_; and (**c**) force method for measuring the limit angle, φ_lim_.

This test method (Figure 7) is not standardized and can be performed at the end of the standard test [14].

At the end of the slide along the plane, the slide is held by a cable, parallel to the plane, and the constraint force (F), exerted by the cable, is measured using a load cell. From the static balance of forces, it is possible to evaluate a new parameter, defined as the limiting friction angle, φ_lim_, which is given by the following expression:

(6) $$tan\varphi_{lim}=tan\beta -\frac{F(\beta )}{Wcos\beta }$$

where *W* is the weight of the box.

Even if the inclination of the plane continues to vary, the constraint reaction exerted by the cable changes in such a way that φ_lim_ remains constant, thus allowing us to obtain another measure of the interface friction in the absence of viscous resistance to the movement and after the first detachment of the box. The parameter φ_lim_ is less than φ_0_ both in the gradual and the sudden sliding modes of failure.

- We would like to reiterate that, in addition to the evaluation of resistance in static conditions, there is a whole area related to dynamic conditions, such as those that can occur in seismic situations, which can be studied with tests that differ from those described up to now. Though this aspect is beyond the scope of this paper, we refer readers to the widespread research on this topic in the literature [15,16,17,18,19,20].

**Figure 8:**
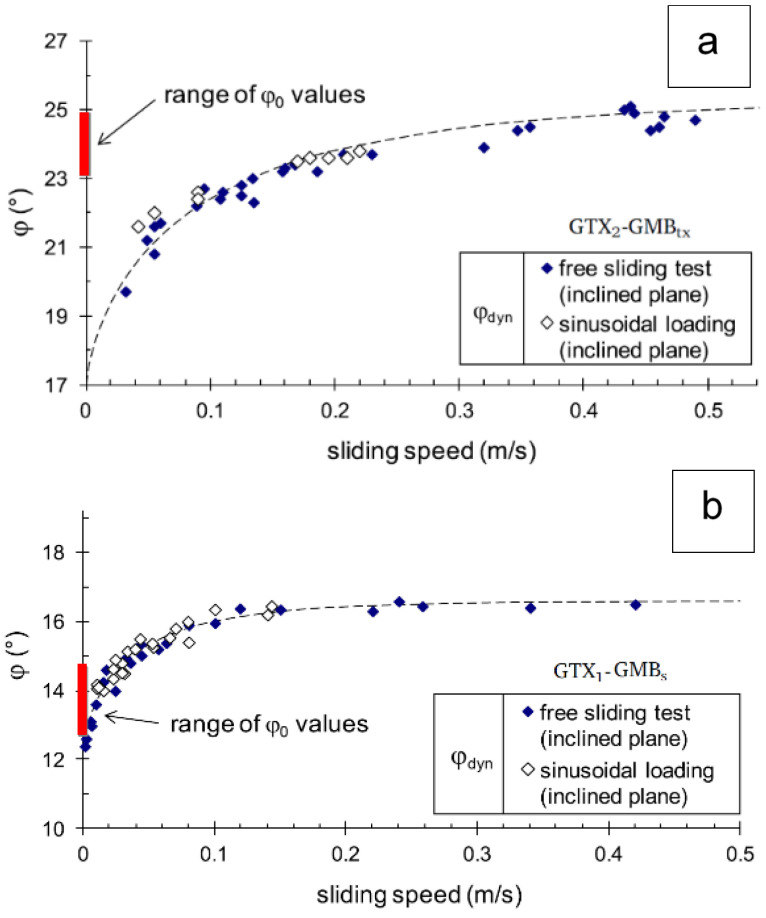
Dynamic friction expressed as a function of the relative sliding velocity for two different HDPE geomembranes in contact with nonwoven geotextiles [19]: (**a**) interface with sudden sliding behaviour; (**b**) interface with gradual sliding behaviour.

**Figure 9:**
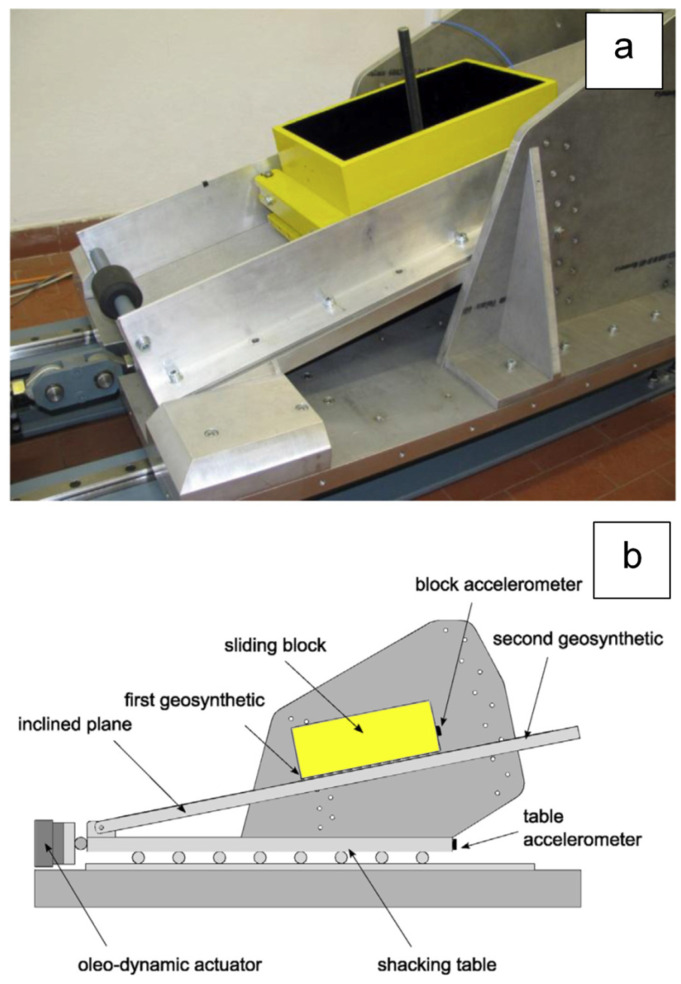
Inclined plane mounted on a vibrating table, available at the geotechnical laboratory of the University of Padua: (**a**) lateral view; (**b**) operational scheme.

Figure 8 [19] reports the relationship between interface friction and slip speed for two interfaces (one of the sudden type, and the other of the gradual type), relating to two different HDPE geomembranes in contact with nonwoven geotextiles, in dry conditions. To establish such a relationship, both inclined plane tests and vibrating table tests (Figure 9) have been carried out.

**Figure 10:**
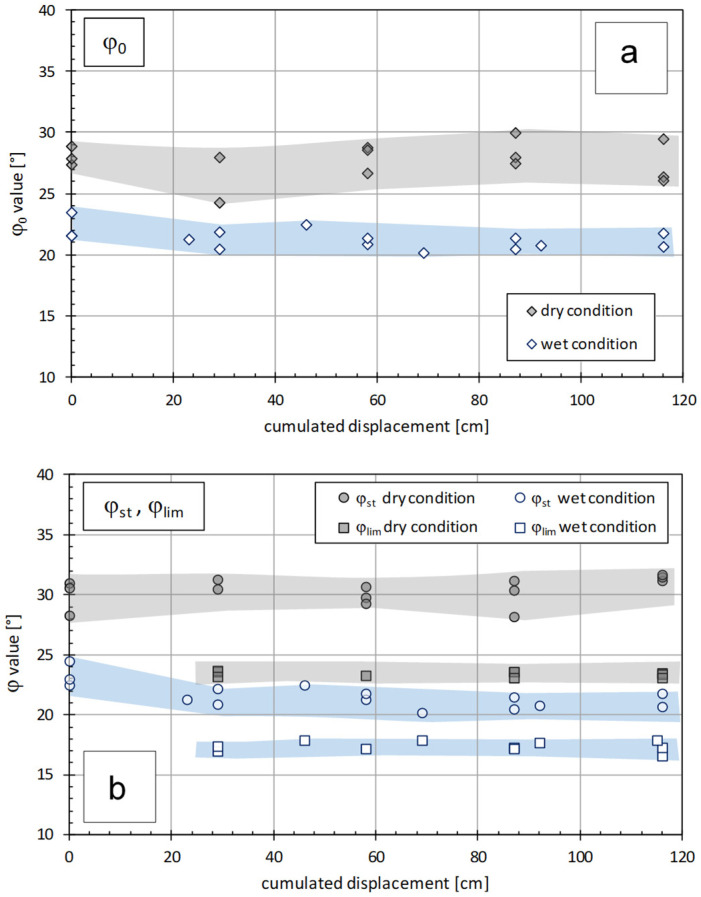
Friction variation for a GCD-GCL interface between a drainage geocomposite and the woven side of a geocomposite clay liner [22]: (**a**) ranges of φ_0_ values versus cumulative displacements for both dry and wet conditions; (**b**) ranges of φ_stand_ and φ_lim_ values versus cumulative displacements for both dry and wet conditions.

- The inclined plane tests also allow one to carry out experiments on the variation in the interface friction between geosynthetics for large relative displacements [21]. Figure 10 reports the results of inclined plane tests at large displacements for an interface between a drainage geocomposite and the woven side of a geocomposite clay liner (GCD-GCL), showing gradual sliding behaviour both in wet and dry conditions [22]. It is possible to note how the presence of fluid at the interface causes a lower frictional resistance, and it is confirmed that the parameter φ_lim_ always remains lower than the corresponding φ_0_ and φ_stand_ values in both the dry and wet conditions.

As already mentioned, geosynthetic landfill cover on a slope may require reinforcements to increase stability. For practical reasons, a geogrid, or a reinforced geomat, is placed immediately over the drainage geocomposite and, subsequently, covered with soil. In these cases, among the various interfaces to be considered for stability analyses, there is also an interface between the geogrid, or the reinforced geomat, and the drainage geocomposite, which is a mixed-type interface since some soil is also present between the two geosynthetics.

While many studies on both geosynthetic–geosynthetic interfaces [23,24,25] and soil–geosynthetic interfaces [26,27,28,29] are available, there is a lack information on hybrid interfaces available in the literature. Recently, some preliminary results obtained at the geotechnical laboratory of the University of Padua using the prototype of direct shear at constant loading speed have been presented [30]. This investigation examined the contact between a geogrid and a drainage geocomposite (GGR-GCD), as well as the contact between a reinforced geomat and the same drainage geocomposite of the previous case (GMT-GCD). For comparison, the interfaces were tested without soil, as well as with soil compacted at different degrees of densification, in order to investigate the influence of the covering soil on the shear strength of a draining-type interface. Both interfaces were tested at a normal stress of about 5 kPa.

As already stated, the friction angle was based on the more realistic and cautious displacement of 1 mm due to the gradual sliding behaviour of both interfaces. In the absence of soil, it was measured as φ_0_ = 25° for GGR-GCD and φ_0_ = 13° for the GMT-GCD interface.

**Figure 11:**
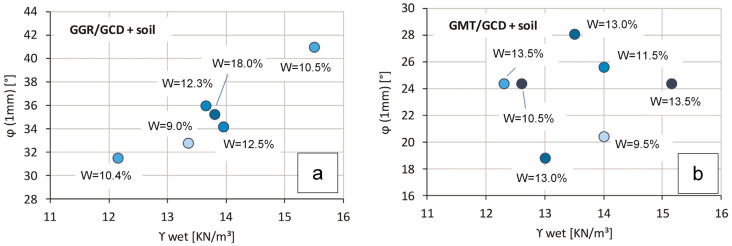
Hybrid interface: friction angle as a function of wet soil density and water content [30]. (**a**) GGR-GCD interface; (**b**) GMT-GCD interface.

A graphical summary of the results obtained when the soil was crammed above both the geogrid and the geomat is shown in Figure 11, wherein data are reported in terms of the shear strength angle, φ_0_, as a function of both the soil unit weight and the water content.

It is observed that the two interfaces reveal different behaviours with respect to the compaction level. In the case of GGR-GCD contact, a correlation with the soil unit weight can be observed, with the friction angle increasing as the soil density increases (Figure 11a). In contrast, in the case of GMT-GCD contact, significant dispersion of data is observed (Figure 11b).

It emerges that in the case of the GGR-GCD interface, characterized by a large opening morphology, the soil is able to increase the friction angle compared to the case without soil, and the sliding kinematics also change from gradual to sudden. Therefore, in this case, the interface strength may depend on the level of compaction.

Conversely, in the case of the GMT-GCD interface, a smaller amount of soil passes through the geomat and comes into contact with the drainage geocomposite. Consequently, even though a general increase in the friction angle is observed, no modification occurs in the sliding mechanism, which remains of the gradual sliding type, and the level of compaction does not significantly influence the friction angle.

## 3. Conclusions

Based on the discussed data, it can be inferred that the correct characterization of the interface strength between the geosynthetics employed in a landfill cover can be achieved using laboratory equipment at low contact stresses, such as the inclined plane and the direct shear at constant speed of loading. With such equipment, it is possible to observe the interface failure modes, sudden sliding, gradual sliding, and non-uniform sliding, and establish which is the most suitable shear strength angle, between φ_0_, φ_stand_, and φ_lim_, in order to guarantee the level of safety with respect to the slipping risk on the slope and to the tear due to the differential settlements that may occur in different areas of the plant.

Similar recommendations can also be extended to the case of seismic loading for the prediction of post-seismic displacements of the cover and/or tensile force induced in the geosynthetics by the displacements themselves.

Given the low level of contact stresses, it is emphasized that the design parameter of interface resistance should be more accurately represented by the secant value, rather than the tangent one, which is usually supplied by the standard direct shear test.

Finally, the standard methodology for establishing interface failure may be acceptable when the kinematic behaviour is non-uniform, so that loading at constant speed of displacement with the standard direct shear test can be considered convenient even if not fully appropriate. Conversely, interface failure showing both uniform and uniformly accelerated motions should be characterized by the angles φ_0_ or φ_lim_, depending on the level of safety to be achieved.

## Data Availability

The raw data supporting the conclusions of this article will be made available by the authors on request.

## References

[1] (2021). Standard Test Method for Determining the Shear Strength of Soil-Geosynthetic and Geosynthetic-Geosynthetic Interfaces by Direct Shear.

[2] (2018). Geosynthetics—Determination of Friction Characteristics—Part 1: Direct Shear Test.

[3] Bacas B.M., Konietzky H., Berini J.C., Sagaseta C. (2011). A new constitutive model for textured geomembrane/geotextile interfaces. Geotext. Geomembr..

[4] Cazzuffi D., Recalcati P., Calvarano L.S. (2023). Direct Shear and Inclined Plane Experimental Activities for Different Interfaces among Geosynthetics and Soils. Geo-Congress 2023.

[5] Pavanello P., Carrubba P., Moraci N. (2022). Evaluation of geosynthetic interface friction at low normal stress: Comparison between two different test procedures. Acta Sci. Pol. Archit..

[6] Pavanello P., Carrubba P., Moraci N. (2022). Geosynthetic interface friction at low normal stress: Two approaches with increasing shear loading. Appl. Sci..

[7] Lalarakotoson S., Villard P., Gourc J.P. (1999). Shear strength characterization of geosynthetic interfaces on inclined planes. Geotech. Test. J..

[8] Reyes Ramirez R., Gourc J.P. (2003). Use of the inclined plane test in measuring geosynthetic interface friction relationship. Geosynth. Int..

[9] Pitanga H.N., Gourc J.P., Vilar O.M. (2009). Interface shear strength of geosynthetics: Evaluation and analysis of inclined plane tests. Geotext. Geomembr..

[10] (2005). Geosynthetics—Determination of Friction Characteristics—Part 2: Inclined Plane Test.

[11] Gourc J.P., Reyes Ramirez R. (2004). Dynamics-based interpretation of the interface friction test at the inclined plane. Geosynth. Int..

[12] Pavanello P., Carrubba P., Moraci N. (2021). The characterisation of geosynthetic interface friction by means of the inclined plane test. Geotext. Geomembr..

[13] Briançon L., Girard H., Gourc J.P. (2011). A new procedure for measuring geosynthetic friction with an inclined plane. Geotext. Geomembr..

[14] Carbone L., Gourc J.P., Carrubba P., Pavanello P., Moraci N. (2015). Dry friction behaviour of a geosynthetic interface using inclined plane and shaking table tests. Geotext. Geomembr..

[15] Kavazanjian E. Current issues in seismic design of geosynthetic cover systems. Proceedings of the 6th International Conference on Geosynthetics.

[16] Kavazanjian E., Matasovic N., Caldwell J. Seismic design and performance criteria for landfills. Proceedings of the 6th US National Conference on Earthquake Engineering.

[17] Kavazanjian E., Wu X., Arab M., Matasovic N. (2018). Development of a numerical model for performance-based design of geosynthetic liner systems. Geotext. Geomembr..

[18] Kim J., Riemer M., Bray J.D. (2005). Dynamic properties of geosynthetic interfaces. Geotech. Test. J..

[19] Pavanello P., Carrubba P., Moraci N. (2018). Dynamic friction and the seismic performance of geosynthetic interfaces. Geotext. Geomembr..

[20] Pavanello P., Carrubba P., Moraci N. (2018). The determination of interface friction by means of vibrating table tests. Geotext. Geomembr..

[21] Pinho-Lopes M., Paula A.M., Lopes M.L. (2016). Soil–geosynthetic interaction in pullout and inclined-plane shear for two geosynthetics. Geosynth. Int..

[22] Pavanello P., Carrubba P., Moraci N., Pezzano P., Miuzzi M. Parameters and conditions affecting friction angles in geosynthetic interfaces. Proceedings of the Eurogeo 6.

[23] Frost J.D., Lee S.W. (2001). Microscale study of geomembrane-geotextile interactions. Geosynth. Int..

[24] Stark T.D., Niazi F.S., Keuscher T.C. (2015). Strength envelopes from single and multi geosynthetic interface tests. Geotech. Geol. Eng..

[25] Bacas B.M., Cañizal J., Konietzky H. (2015). Shear strength behaviour of geotextile/geomembrane interfaces. J. Rock Mech. Geotech. Eng..

[26] Moraci N., Cardile G., Gioffré D., Mandaglio M.C., Calvarano L.S., Carbone L. (2014). Soil geosynthetic interaction: Design parameters from experimental and theoretical analysis. Transp. Infrastruct. Geotechnol..

[27] Palmeira E.M. (2009). Soil-geosynthetic interaction: Modelling and analysis. Geotext. Geomembr..

[28] Ferreira F.B., Vieira C.S., Lopes M.L. (2015). Direct shear behaviour of residual soil–geosynthetic interfaces—Influence of soil moisture content, soil density and geosynthetic type. Geosynth. Int..

[29] Ferreira F.B., Fernandes J., Vieira C.S., Lopes M.L. Soil-geosynthetic interface shear behaviour: Insights from inclined plane and direct shear tests. Proceedings of the 12th International Conference on Geosynthetics.

[30] Pavanello P., Carrubba P. The effect of soil on the shear strength of geosynthetic interfaces. Proceedings of the 12th International Conference on Geosynthetics.

